# Melatonin Alleviates Intestinal Barrier Damaging Effects Induced by Polyethylene Microplastics in Albino Rats

**DOI:** 10.3390/ijms241713619

**Published:** 2023-09-03

**Authors:** Walaa Bayoumie El Gazzar, Rania E. Sliem, Heba Bayoumi, Hend Elsayed Nasr, Manar Shabanah, Amira Elalfy, Shaimaa E. Radwaan, Mohammed A. Gebba, Heba M. Mansour, Amul M. Badr, Marwa Fathy Amer, Sara S. Ashour, Heba Morsi, El Shaimaa Ahmed Fahmy Aboelkomsan, Bodour Baioumy, Alaa El-Din Hamid Sayed, Amina A. Farag

**Affiliations:** 1 Department of Anatomy, Physiology and Biochemistry, Faculty of Medicine, The Hashemite University, P.O. Box 330127, Zarqa 13133, Jordan; 2 Department of Medical Biochemistry and Molecular Biology, Faculty of Medicine, Benha University, Benha 13518, Egypt; hend.mosalm@fmed.bu.edu.eg; 3 Department of Zoology, Faculty of Science, Benha University, Benha 13518, Egypt; rania.elsayed@fsc.bu.edu.eg (R.E.S.); shaimaa.elsayed@fsc.bu.edu.eg (S.E.R.); 4 Department of Histology and Cell Biology, Faculty of Medicine, Benha University, Benha 13518, Egypt; heba.bayoumi@fmed.bu.edu.eg (H.B.); amira.alalfay@fmed.bu.edu.eg (A.E.); 5 Department of Physiology, Faculty of Medicine, Mansoura University, Mansoura 35511, Egypt; manar.shabana@yahoo.com; 6 Department of Anatomy and Embryology, Faculty of Medicine, Benha University, Benha 13518, Egypt; mohammedgebba@gmail.com (M.A.G.);; 7 Department of Anatomy and Embryology, Faculty of Medicine, Merit University, Sohag 82524, Egypt; 8 Department of Pharmacology and Toxicology, College of Pharmaceutical Sciences and Drug Manufacturing, Misr University for Science and Technology, 6th of October City 12573, Egypt; heba.soliman@must.edu.eg; 9 Department of Medical Biochemistry and Molecular Biology, Faculty of Medicine, Cairo University, Cairo 11451, Egypt; amal.abdelrehem@kasralainy.edu.eg (A.M.B.); marwa.fathy@kasralainy.edu.eg (M.F.A.); sara.salama@kasralainy.edu.eg (S.S.A.); hebamorsi@kasralainy.edu.eg (H.M.); 10 Department of Pathology, School of Medicine, New Giza University (NGU), Giza 12573, Egypt; 11 Department of Zoology, Faculty of Science, Assiut University, Assiut 71516, Egypt; alaasayed@aun.edu.eg; 12 Department of Forensic Medicine and Clinical Toxicology, Faculty of Medicine, Benha University, Benha 13518, Egypt; amina.farag@fmed.bu.edu.eg

**Keywords:** polyethylene microplastics, intestinal barrier, tight junction proteins, melatonin, proinflammatory cytokines

## Abstract

There have been concerns about the potential health risks posed by microplastics (MP). The detection of MP in a variety of food products revealed that humans are ingesting MP. Nevertheless, there is a paucity of data about their impacts, as well as their uptake, on intestinal barrier integrity. This study examined the toxic effects of oral administration of two doses of polyethylene microplastics (PE-MP) (3.75 or 15 mg/kg/day for 5 weeks; mean particle size: 4.0–6.0 µm) on the intestinal barrier integrity in rats. Moreover, the effect of melatonin treatment with MP exposure was also assessed. The PE-MP particle uptake, histopathological changes, Alcian blue staining, Muc2 mRNA, proinflammatory cytokines (IL-1β and TNF-α), and cleaved caspase-3, as well as tight junction proteins (claudin-1, myosin light-chain kinase (MLCK), occludin, and zonula occludens-1 (ZO-1)) were assessed. Oral administration of PE-MP resulted in apparent jejunal histopathological alterations; significantly decreased mucin secretion, occludin, ZO-1, and claudin-1 expression; and significantly upregulated MLCK mRNA, IL-1β concentration, and cleaved caspase-3 expression. Melatonin reversed these altered parameters and improved the PE-MP-induced histopathological and ultrastructure changes. This study highlighted the PE-MP’s toxic effect on intestinal barrier integrity and revealed the protective effect of melatonin.

## 1. Introduction

MP pollution is now considered among the world’s most significant environmental issues [1]. It refers to thin films, particles, or plastic debris with a diameter < 5 mm [2]. Polyethylene (PE) is the most prevalent type utilized in houseware, pipes, bottles, toys, food packing films, containers, reusable bags, and trays [3]. Because of the compact size of MP, it is readily ingested by organisms and accumulates in vivo, which is biologically hazardous [4]. The identification of MP in numerous dietary products confirmed human MP ingestion. MP is ubiquitous in wastewater, groundwater, and surface water. Plastics have been detected in drinking water; therefore, this problem has been examined recently [5,6]. Numerous studies have proven that ingested MP accumulates in the gut of numerous species [7,8,9].

Epithelial organs, including gastrointestinal tracts, are subjected to various assaults from chemical, biological, or pathogenic insults. Nonetheless, the organism maintains these barriers’ integrity in some cases, thereby preventing chronic inflammation. In addition to forming a physical layer, several epithelial, as well as nonepithelial, cell types produce multilayered, biochemical, highly dynamic physical and immunological protection to preserve tissue homeostasis [10]. This barrier system must be permeable selectively in order to permit nutrients and water absorption while continually preventing harmful noxae. The barrier is often intact, preventing the initiation of an uncontrolled inflammatory response. Nevertheless, the barrier is compromised in some situations, causing an inflammatory reaction to eject the invading noxae [11].

The intestinal barrier consists of several layers. The outer layer includes secretory immunoglobulin A (sIgA), mucus, defense proteins like antimicrobial proteins (AMPs), and the commensal gut microbiota. The middle layer consists of intestinal epithelial cells (IECs), whereas the inner part comprises immune cells of adaptive, as well as innate, immunity [10]. Tight junctions (TJs) between adjacent intestinal epithelia are vital for the functioning of the physical intestinal barrier. The tight junction consists of numerous cytosolic and transmembrane proteins, including junctional adhesion molecules (JAM), cingulin, occludin, zonula occludens (ZOs), claudins, and tricellulin, interacting with each other, as well as with the cytoskeleton, and build a complex architecture [12].

The claudins form the backbone of tight junctions and are the most important components of the tight junctions. Occludins are important in maintaining the stability and barrier function of the tight junctions [13]. Occludin expression has been found to be severely reduced in disease models of intestinal inflammatory disorders, implying that it plays an important role in barrier integrity maintenance [12]. ZO-1 is also assumed to function as a multidomain scaffold, coordinating the assembly of transmembrane and cytosolic proteins into the tight junction and/or regulates the activity of these proteins once assembled [14]. Myosin light chain kinase (MLCK) is a Ca^2+^ calmodulin-dependent serine/threonine kinase that constantly modulates actomyosin reorganization and cell contraction. MLCK is involved in intestinal epithelial regulation, inflammation, and gastrointestinal disorders [15]. The phosphorylation of myosin light chain (MLC) by MLCK results in increased paracellular permeability and epithelial shedding at villous tips [16]. 

MP with dimensions greater than 150 μm are not absorbed. They remains bound to the intestinal mucus layer and directly interact with intestinal epithelial cells’ apical parts [17]. Numerous studies on how MP impact on the gut tract demonstrated that they could result in oxidative stress and inflammation in intestinal epithelial cells, alternations in intestinal permeability, alternations in mucin’s volume and expression, alternations in gut microbiota composition, and resulting in immune cell recruitment or changing cytokine secretion [18,19]. According to the findings of Li et al. (2020), PE-MP can cause intestinal dysbacteriosis and inflammation [20]. Jia et al. (2023) reported that polypropylene MP induced colonic redox system imbalance, inflammatory reactions, decreased mucus secretion and damaged the colonic tight junctions [21]. PVC-MPs also reduced intestinal mucus output and increased intestinal permeability, according to Chen et al. (2022). Furthermore, they demonstrated significant alterations in gut microbiome composition and metabolome profiles [22]. On the contrary, some studies revealed no oxidative stress, inflammation, or other adverse health effects [23]. Moreover, whether MP affect the intestinal barrier integrity through their impact on the intestinal epithelial tight junction proteins is not well clarified.

Melatonin is a hormone generated by the pineal gland controlling circadian rhythm, as well as other hormones. It has been proven to have several therapeutic effects, such as immunoregulation, anti-inflammatory, and antioxidant properties [24,25,26,27]. Melatonin treatment has also improved GI tract diseases [28,29,30,31]. Melatonin administration, according to Ahmed et al. (2022), may be effective in the prevention of experimental colitis in rats due to its antioxidant and anti-inflammatory effects [28]. Kim et al. (2020) reported a link between melatonin and microbiota, demonstrating that bacterial sensing via TLR4, mucin, and Reg3 production by goblet cells was involved in the anti-colitic effects of melatonin, implying that melatonin may be useful in microbiota control and therapeutics for irritable bowel syndrome (IBD) [29]. Melatonin has also been shown to be an effective treatment for improving IBS score, GI symptoms, and quality of life in IBS patients due to its anxiolytic, anti-inflammatory, anti-oxidative, and motility regulation actions [32]. Conversely, some studies illustrated that melatonin immunological actions are not constant; nonetheless, they are conditional and have the potential to ameliorate both pro- and anti-inflammatory impacts [33]. 

PE-MP comprise most MP in the environment. Furthermore, most MP in the environment have a size between 1 and 10 µm [34]. Moreover, several studies have reported that environmental pollutant-induced adverse health effects are generally caused by chronic exposure at low doses [35,36]. With reference to Park et al. (2020), they administered PE-MP to mice via gavage at doses of 3.75, 15, or 60 mg/kg body weight in accordance with a protocol approved by the Korea Institute of Toxicology and proposed that the NOAEL for reproductive and developmental toxicity of PE-MP dosed repeatedly for 90 days is less than 15 mg/kg bw/day [35]. Therefore, in this study, 4–6 µm PE-MP at doses of 3.75 and 15 mg/kg body weight was utilized to determine the impacts of PE-MP oral exposure on the intestinal histopathology, intestinal mucin secretion and epithelial tight junction protein expressions as essential components of the intestinal barrier function. Additionally, we attempted to determine whether melatonin treatment could effectively alleviate any intestinal damage expected from PE-MP exposure. 

## 2. Results

There were no substantial differences between subgroup IA and subgroup IB (control groups) in all studied parameters.

### 2.1. PE-MP Particles Uptake by the Jejunum

**Figure 1:**
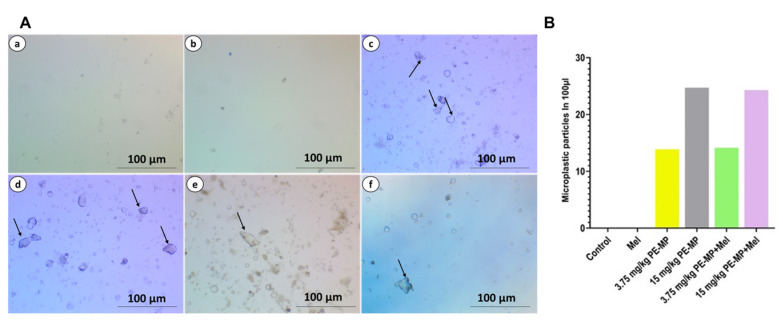
(**A**) Representative photographs of light images of PE-MP particles in the jejunal tissue samples from experimental groups: (**a**) control group; (**b**) melatonin group; (**c**) 3.75 mg/kg PE-MP; (**d**) 15 mg/kg PE-MP; (**e**) 3.75 mg/kg PE-MP + melatonin; (**f**) 15 mg/kg PE-MP + melatonin. (**B**) Quantification of PE-MP particles in the jejunal tissue samples after digestion in hydrogen peroxide.

The PE-MP particle uptake by the jejunum was demonstrated and quantified by light microscopic images, which confirmed substantially higher numbers of PE-MP particles in PE-MP-exposed groups relative to controls in a dose-dependent manner (Figure 1). 

### 2.2. Melatonin Improved Jejunal Histopathological Changes Induced by PE-MP Particles

**Figure 2:**
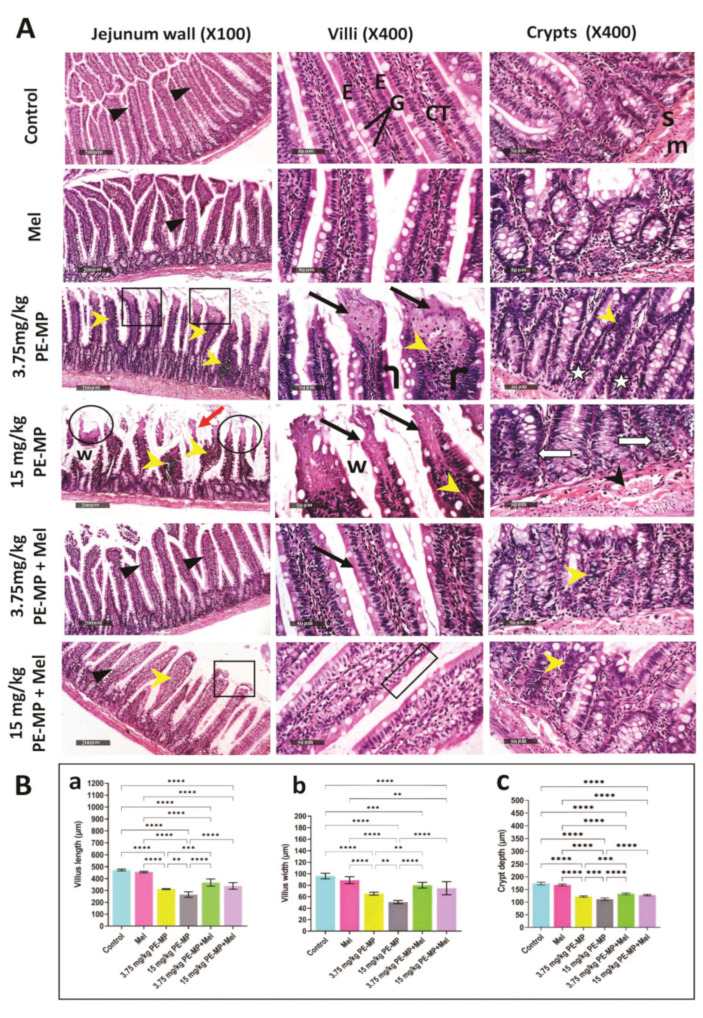
(**A**) A representative set of H&E-stained jejunum sections from experimental groups. Control group: The jejunal wall is formed of intact mucosa with intact villi and crypts, submucosa (s), and external muscle coat (m). The villi appear as leaf-like projections (triangles) lined by enterocytes (E) with basal, oval, euchromatic nuclei, and abundant mucous-secreting goblet cells (G). Each villus has a connective tissue core (CT). Mel-treated group: showed almost the same records as the control samples without abnormal histological changes. 3.75 mg/kg PE-MP-treated group: The villi have marked mucosal surface erosions (squares) with remarkable necrotic tips (black arrows). The lining epithelium showing pyknotic nuclei (bent arrows). The CT cores have massive inflammatory cell infiltration (yellow arrowheads). The crypts are displaying notable hyperchromatic nuclei of the lining cells and apparent decrease of the well differentiated goblet cells (stars). 15 mg/kg PE-MP-treated group: Showed almost the same finding as the previous group but more exaggerated. The villi are shortened with atrophied tips (circles) and lost epithelial lining (black arrows). The inter-villous spaces are widened (w) and are filled with sloughed tissue (red arrow). The crypts have vacuolated cell lining (white arrows) with apparent decrease of the well differentiated goblet cells. Congested vasculature are apparent in the submucosa (black arrowhead). 3.75 mg/kg PE-MP + Mel-treated group: Apparent protective efficacy on intestinal villi covering epithelium integrity without abnormal erosion (triangles). The villi show almost normal histology (arrows). Some crypts are showing mild inflammatory cell infiltrate (yellow arrowheads). 15 mg/kg PE-MP + Mel-treated group: The histological appearance has improved (triangles), but a few villi still have necrotic tips (square) and focal atrophied lining epithelium (rectangle). Mild inflammatory cells (yellow arrowheads) still apparent in the crypts. (Jejunum wall: scale bar = 200 μm, magnification = ×100; villi and crypts: scale bar = 50 μm, magnification = ×400.) (**B**) Histograms represent: (**a**) villous length (μm); (**b**) villous width (μm); (**c**) crypt depth (μm) in all experimental groups. The results are expressed as the mean ± SD. ** Significant at *p* ˂ 0.01; *** *p* ˂ 0.001, and **** *p* < 0.0001.

As demonstrated in Figure 2, control groups exhibited a normal morphological structure of the jejunal intestinal wall as detected in the apparent intact intestinal mucosa, including crypts and villi with their abundant goblet cells and lining enterocytes, as well as outer muscular coat and intact submucosa. Exposure to PE-MP induced apparent villous degeneration and shortening with epithelial atrophy, marked inflammatory cell infiltration of the lamina propria, vacuolated crypt epithelial lining, widened inter-villous spaces, and congested submucosal blood vessels.

Rats treated with melatonin showed improved pathological changes with nearly normal villous and crypt morphology. This improvement was confirmed statistically by a significant increase in villous height, width, and crypt depth (Figure 2).

### 2.3. Melatonin Improved the Enterocytes Ultrastructure Pathological Changes Induced by the PE-MP Particles

**Figure 3:**
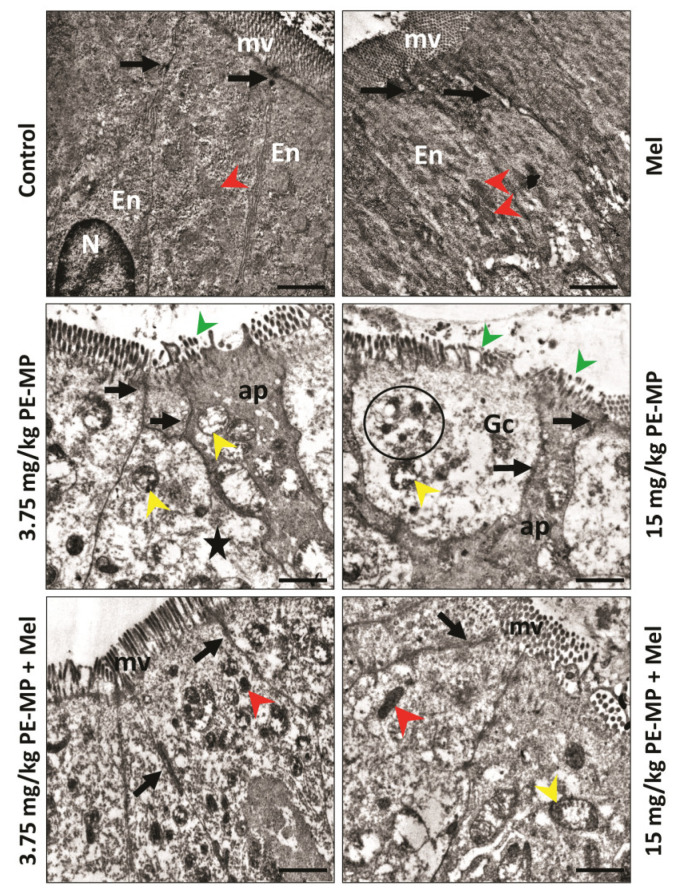
A representative set of electron micrographs of enterocytes from jejunum sections from experimental groups. Control group: Normal tall columnar enterocytes (En) with oval basal nuclei (N), mitochondria (red arrowheads), and finger-like apical projecting microvilli (mv). The apical parts of the lateral membranes display dense areas of the tight junction (black arrows) between cells. Mel-treated group: has the same normal histological morphology. 3.75 mg/kg PE-MP-treated group: The enterocytes appeared necrotic with rarified cytoplasm (star), swollen mitochondria with destructed cristae (yellow arrowheads), and broken apical microvilli (green arrowhead). Some apoptotic cells (ap) with electron-dense cytoplasm appeared. Notice the decreased density of the cellular junctions between cells (black arrows). 15 mg/kg PE-MP-treated group: Showed intensified previous group results. Moreover, some enterocytes became like ghost cells (GCs) with nuclear karyolysis (circle). 3.75 mg/kg PE-MP + Mel- and 15 mg/kg PE-MP + Mel-treated groups: Showed improved histological ultrastructure, with nearly normal tight junctions. Some mitochondria appeared normal (red arrowheads), while others still degenerated with destructed cristae (yellow arrowheads). (Scale bar = 2 μm, direct magnification = ×10,000).

The ultrastructure of the control groups displayed normal columnar enterocytes with basal nuclei, normal mitochondria, and apical microvilli. The cells were closely opposed to each other due to the junctional complexes between the cells. The PE-MP-treated groups displayed necrotic enterocytes with rarified cytoplasm, degenerated mitochondria, broken microvilli, and affected tight junctions. Some apoptotic cells were apparent. Some enterocytes turned into ghost cells with karyolysis nuclei in the 15 mg/kg PE-MP-exposed group (Figure 3). Melatonin therapy apparently improved the enterocytes’ histological ultrastructure, with nearly normal microvilli and tight junctions. However, some mitochondria appeared normal while others still degenerated with destructed cristae (Figure 3).

### 2.4. Impact of PE-MP Exposure and Melatonin Treatment on the Mucin Secretion and Muc2 mRNA Expression

**Figure 4:**
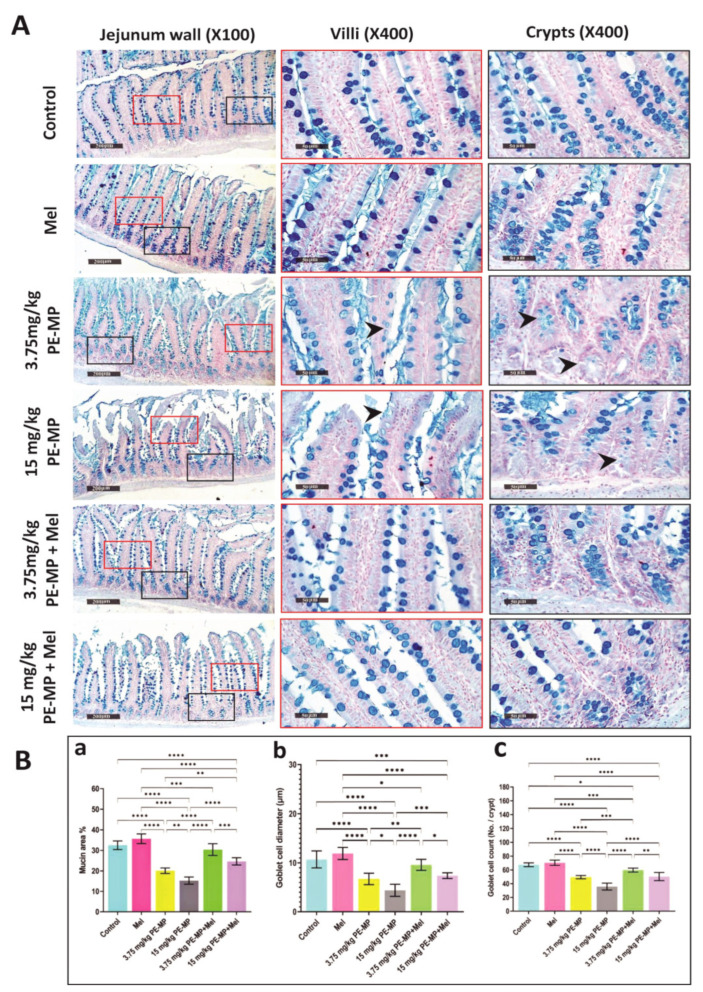
(**A**) Representative set of Alcian-blue-stained jejunum sections from experimental groups. The blue-stained areas represent mucin expression (1st column represents low magnification of the full jejunal thickness of the experimental groups while, the 2nd and the 3rd columns represent magnified red boxed regions (villi) and black boxed regions (crypts), respectively). Control group: intense Alcian-blue-stained mucin areas in goblet cells and intestinal lumina of both villi and crypts. Mel-treated group: shows nearly the same mucin distribution as the control group. PE-MP-treated groups shows a marked reduction in Alcian-blue-stained areas (arrowheads) compared to the control group indicating a defective intestinal mucous barrier. The reduced mucin was dose dependent (higher in the 15 mg/kg PE-MP- than the 3.75 mg/kg PE-MP-treated group). Melatonin protected the intestinal mucosal barrier, as indicated by considerably increased mucin in the 3.75 mg/kg PE-MP + Mel- and 15 mg/kg PE-MP + Mel-treated groups. (Intestinal wall: scale bar = 200 μm, magnification = ×100; villi and crypts: scale bar = 50 μm, magnification = ×400.) (**B**) Histograms represent: (**a**) mucin area %; (**b**) goblet cell diameter (μm); (**c**) goblet cell count (no/crypt) in all experimental groups. The results are expressed as the mean ± SD. * Significant at *p* ˂ 0.05; ** *p* ˂ 0.01; *** *p* ˂ 0.001, and **** *p* < 0.0001.

**Figure 5:**
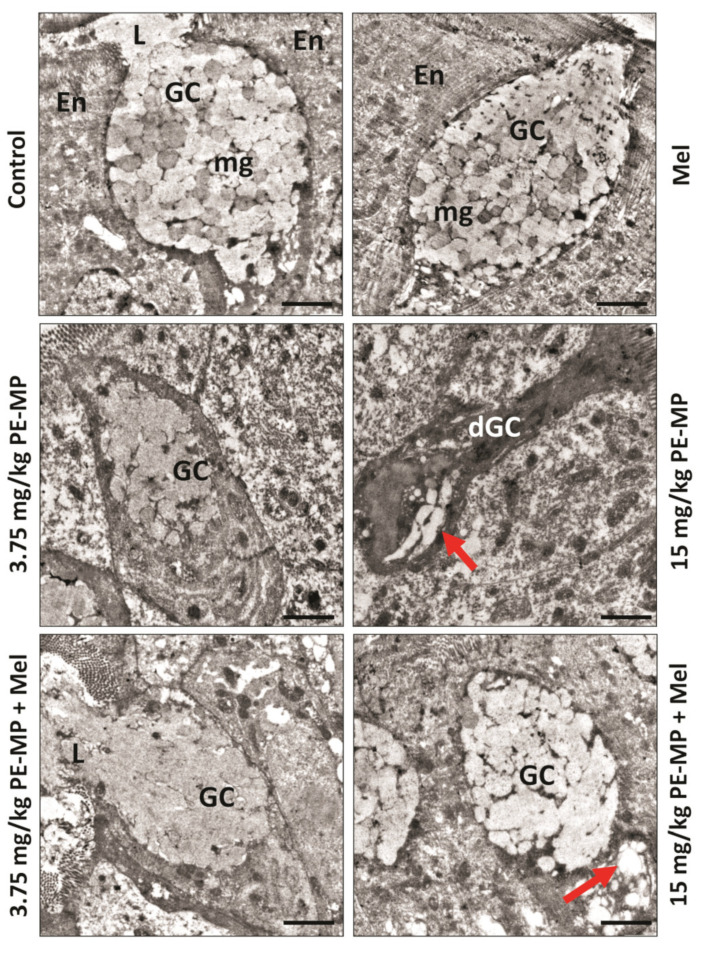
A representative set of electron micrographs of goblet cells from jejunum sections from experimental groups. Control group: Normal goblet cell (GC) between enterocytes (En). Each goblet cell has a narrow base and a wide apex. The cytoplasm is filled with numerous mucin granules (mg). Goblet cells empty their secretions in the intestinal lumen (L). Mel-treated group: demonstrated the same normal histological morphology. 3.75 mg/kg PE-MP-treated group: Goblet cells appeared with fewer mucin granules, while the 15 mg/kg PE-MP-treated group exhibited degenerated goblet cells (dGC) with dark cytoplasm showing lytic foci (red arrow) and nearly absent mucin granules. 3.75 mg/kg PE-MP + Mel- and 15 mg/kg PE-MP + Mel-treated groups: showed improved histological ultrastructure of goblet cells with restored secretion, but some cells still had vacuolated cytoplasm (red arrow). (Scale bar = 2 μm, direct magnification = ×10,000.)

**Figure 6:**
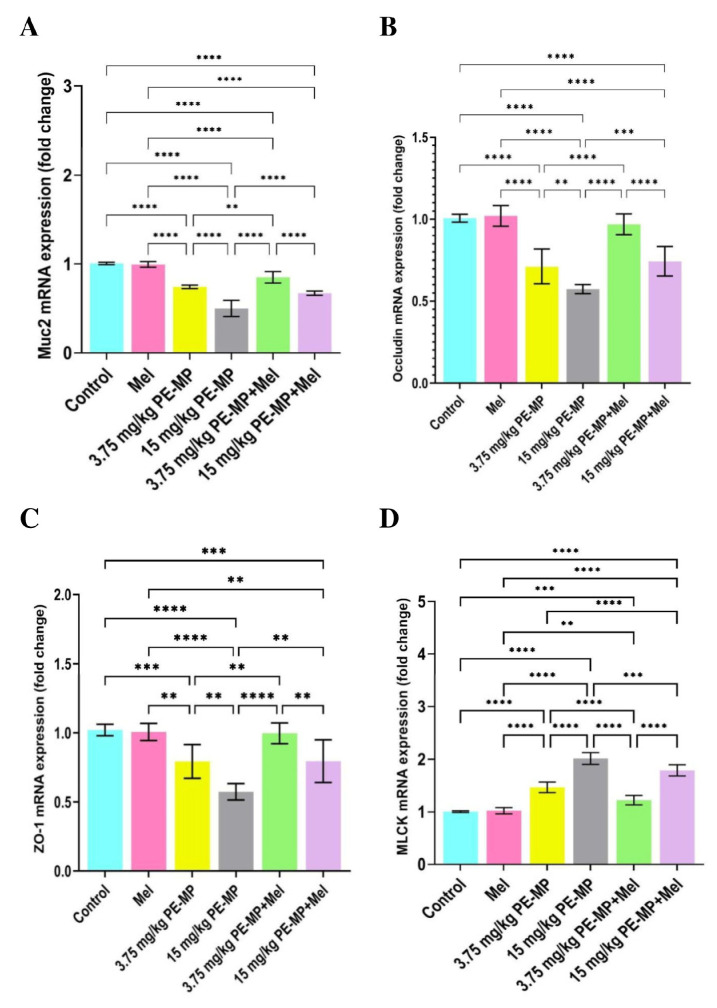
(**A**) Muc2, (**B**) occludin, (**C**) ZO-1, and (**D**) MLCK gene expression in jejunal tissue samples from experimental groups (n = 7/group), as determined by qPCR, normalized for the house-keeping gene B-actin, and expressed relative to controls. Results are expressed as the mean ± SD. ** Significant at *p* ˂ 0.01, *** *p* ˂ 0.001, and **** *p* < 0.0001.

As shown in Figure 4A, Alcian blue staining demonstrated that mucin secretion substantially declined following exposure to 3.75 and 15 mg/kg of PE-MP compared with the control. Mucin area %, goblet cell count, and goblet cell diameter analysis further illustrated the decrease in the mucin secretion as they displayed a significant decrease in the 3.75 and 15 mg/kg PE-MP-treated groups compared to controls. A marked decline in the mucin area %, goblet cell diameter, and goblet cell count was also demonstrated in the 15 mg/kg PE-MP-exposed group compared to the 3.75 mg/kg PE-MP-exposed group (Figure 4B). The electron microscopy (EM) analysis further confirmed the above results as the 3.75 mg/kg PE-MP-exposed group showed decreased mucin granules while the 15 mg/kg PE-MP-exposed group showed degenerated goblet cell and apparently diminished secretion (Figure 5). In addition, Muc2 mRNA’s expression level was substantially downregulated in both the 3.75 and 15 mg/kg PE-MP-treated groups compared with the control. Muc2 mRNA’s transcription levels decreased significantly in the high dose (15 mg/kg) PE-MP-exposed group compared to the low dose (3.75 mg/kg) PE-MP-exposed group (Figure 6A).

Interestingly, melatonin treatment significantly enhanced goblet cells’ measurements and mucin secretion. Mucin secretion demonstrated with Alcian Blue staining, mucin area %, goblet cell count, and goblet cell diameter was significantly increased with melatonin treatment in both the 3.75 PE-MP + melatonin and 15 PE-MP + melatonin groups compared to the 3.75 PE-MP and 15 PE-MP-exposed groups, respectively (Figure 4A,B). The electron microscopy (EM) analysis also showed restored mucin granules and a nearly normal appearance of goblet cells with melatonin treatment (Figure 5). Moreover, Muc2 mRNA’s expression level was significantly elevated in the 3.75 PE-MP + melatonin and 15 PE-MP + melatonin groups compared to the 3.75 PE-MP and 15 PE-MP-exposed groups, respectively (Figure 6A).

### 2.5. Impact of PE-MP Exposure and Melatonin Treatment on the Expression of Intestinal Epithelial Tight Junction Proteins Occludin, ZO-1, Claudin-1, and MLCK

**Figure 7:**
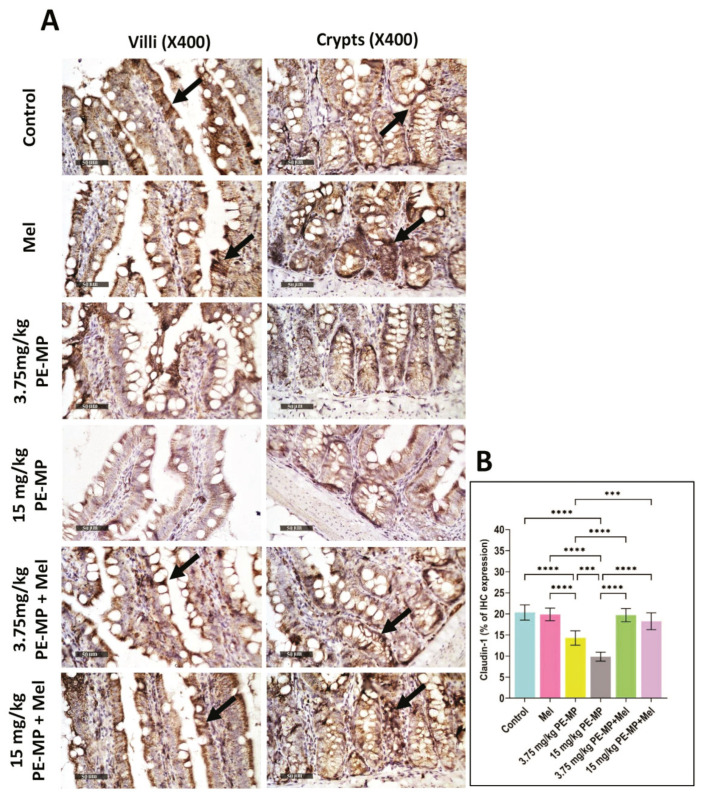
(**A**) A representative set of anti-claudin-1 immune-stained jejunum sections from the experimental groups. The control group and Mel-treated group displayed marked cytoplasmic immune expression of claudin-1 (arrows) in the intestinal epithelial lining of both villi and crypts. The PE-MP-treated groups showed a marked reduction in immune expression compared to the controls indicating loss of intestinal epithelial integrity. The reduced immune expression was dose dependent (higher in the 15 mg/kg PE-MP- than in the 3.75 mg/kg PE-MP-treated group). Mel apparently displayed a protective role detected by elevated claudin-1 immune expression compared to pathological groups. (Scale bar = 50 μm, magnification = ×400). (**B**) The histogram represents claudin-1% immune expression in the experimental groups. Data are presented as the mean ± SD. *** *p* ˂ 0.001, **** *p* < 0.0001.

The results of the qPCR analysis for the mRNA gene expression of occludin (Figure 6B), ZO-1 (Figure 6C), and MLCK (Figure 6D) showed that PE-MP exposure significantly upregulated MLCK and downregulated occludin and ZO-1 mRNA in both the 3.75 and 15 mg/kg PE-MP-exposed groups in comparison with controls. In addition, immunohistochemistry analysis showed significantly decreased immunoreactivity of claudin-1 (Figure 7) in both the 3.75 and 15 mg/kg PE-MP-exposed groups in comparison with controls. Moreover, a substantial elevation in MLCK, and marked declines in occludin, ZO-1, and claudin-1 expressions were detected in the 15 mg/kg PE-MP-exposed group compared to the 3.75 mg/kg PE-MP-exposed group indicating that the effect of PE-MP exposure occurred in a concentration-related manner. 

As shown in Figure 6, melatonin treatment significantly elevated mRNA expression of ZO-1 and occludin as well as downregulated MLCK in both 3.75 PE-MP + melatonin and 15 PE-MP + melatonin groups in comparison to the 3.75 PE-MP and 15 PE-MP-exposed groups, respectively. In addition, PE-MP-induced attenuation in immunoreactivity for claudin-1 was significantly reversed by melatonin treatment (Figure 7). A nearly normal cellular junction was observed by EM examination in melatonin-treated groups (Figure 3).

### 2.6. Effect of PE-MP Exposure and Melatonin Treatment on the Proinflammatory Cytokines (IL-1β and TNF-α)

**Figure 8:**
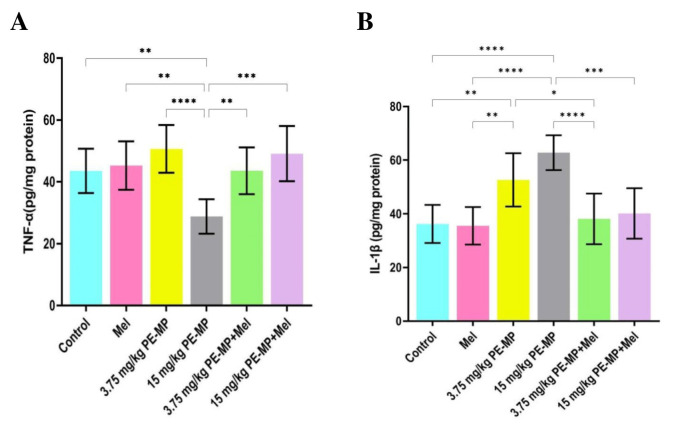
Proinflammatory cytokines levels in jejunal tissue samples from experimental groups (n = 7/group). (**A**) TNF-α and (**B**) IL-1β. Data are expressed as the mean ± SD. * Significant at *p* ˂ 0.05, ** *p* ˂ 0.01, *** *p* ˂ 0.001, and **** *p* < 0.0001. Melatonin impact on proinflammatory cytokines (TNF-α and IL-1β).

IL-1β and TNF-α concentrations were measured in the jejunal tissue samples to determine whether PE-MP exposure could affect the proinflammatory cytokine release. In this study, low-dose (3.75 mg/kg) PE-MP exposure did not result in any alternations in TNF-α concentration in comparison to that in the control. Nevertheless, a significant decrease was demonstrated in the high dose (15 mg/kg) PE-MP-exposed group compared to all studied groups (Figure 8A). Compared to controls, a statistically substantial elevation in IL-1β concentration was detected in the 3.75, and 15 mg/kg PE-MP-exposed groups. An elevation in the IL-1β concentration was detected in the 15 mg/kg PE-MP-exposed group compared to the 3.75 mg/kg PE-MP-exposed group, but this elevation was statistically insignificant (Figure 8B).

Melatonin treatment significantly increased TNF-α concentration in the 15 mg/kg PE-MP+ melatonin group compared to the 15 mg/kg PE-MP-exposed group (Figure 8A). In addition, a substantial decline in the concentration of IL-1β was detected with melatonin treatment in both 3.75 PE-MP + melatonin and 15 PE-MP+ melatonin groups in comparison to the 3.75 mg/kg PE-MP and 15 mg/kg PE-MP-exposed groups, respectively (Figure 8B).

### 2.7. Melatonin Effect on Intestinal Apoptosis Induced by PE-MP Exposure

**Figure 9:**
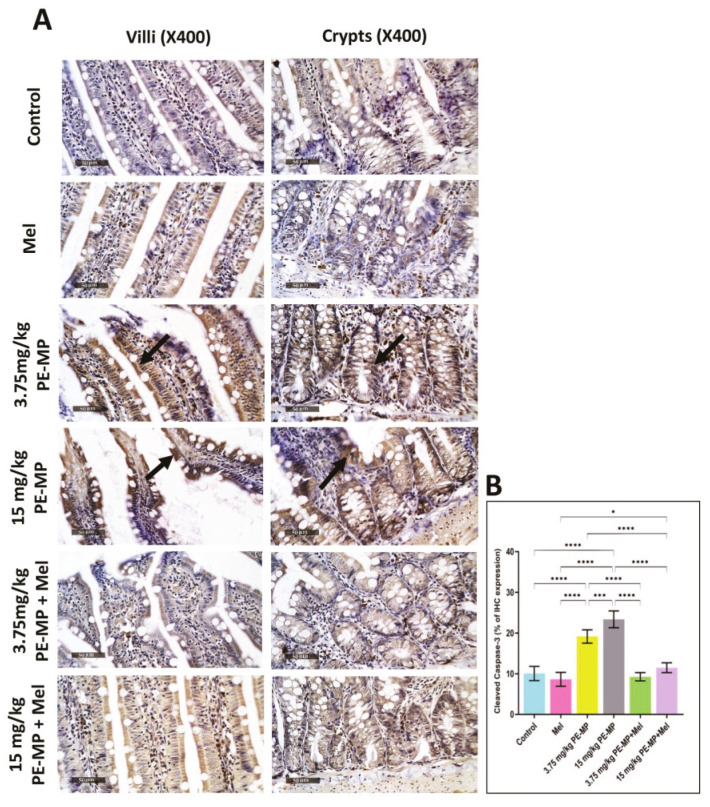
(**A**) A representative set of cleared anti-caspase-3 immune-stained jejunum sections from experimental groups. The control and Mel-treated groups displayed mild cytoplasmic immune expression of cleaved caspase-3. Conversely, PE-MP-treated groups showed intense immune expression (arrows) compared to controls. The increased immune expression was dose dependent (higher in 15 mg/kg PE-MP than 3.75 mg/kg PE-MP-treated group). Mel apparently displayed a protective role detected by decreased cleaved caspase-3 immune expression compared to pathological groups. (Scale bar = 50 μm, magnification = ×400). (**B**) The histogram represents Cleaved caspase-3% expression in the experimental groups. The outcomes are expressed as the mean ± SD. * Significant at *p* ˂ 0.05, *** *p* ˂ 0.001, and **** *p* < 0.0001. Melatonin effect on intestinal apoptosis induced by PE-MP exposure.

Compared to controls, immunostaining for cleaved caspase-3 in jejunal sections revealed significantly increased positive cells in both 3.75 and 15 mg/kg PE-MP-exposed groups. In addition, the 15 mg/kg PE-MP-exposed group displayed significantly increased positive cells compared to the 3.75 mg/kg PE-MP-exposed group (Figure 9). The increased caspase-3 immunoreactivity validated the increased apoptosis signs detected by EM examination (Figure 3).

Melatonin therapy significantly attenuated caspase-3 immunoreactivity in both 3.75 mg/kg PE-MP+ melatonin and 15 mg/kg PE-MP+ melatonin groups compared to the 3.75 mg/kg PE-MP and 15 mg/kg PE-MP-exposed groups, respectively (Figure 9). The antiapoptotic effect of melatonin was further illustrated through decreased apoptosis cells detected by EM examination (Figure 3).

## 3. Discussion

The widespread presence of MP in the environment exposes humans to MP via dermal contact, inhalation, and ingestion [37], posing a significant concern for possible long-term health hazards. Several investigations on many species demonstrated that ingested MP accumulate in the gut of diverse animals; hence, the toxicological implications of chronic MP exposure on intestinal health are currently being evaluated [6]. With oral administration of PE-MP, the gastrointestinal tract may be the major target organ. Mechanical, as well as chemical, barriers are vital components of the intestinal barrier. The mechanical barrier comprises intact intestinal epithelial cells and dense intercellular connections. Mucus is secreted by goblet cells in the intestinal epithelium to create the intestinal mucus barrier, a crucial chemical barrier component [21].

The gastrointestinal mucus layer functions as the frontline defense physiological barrier. Muc2 mucin, which is generated by goblet cells, constitutes the majority of this mucus layer [38]. To assess the impacts of PE-MP exposure on intestinal mucus secretion, Muc2 mRNA gene expression was analyzed as Muc2 is the predominant gel-forming mucin contributing to mucus barrier formation. Muc2 transcription levels were substantially downregulated in the PE-MP-exposed groups compared to controls. Moreover, exposure to 15 mg/kg PE-MP induced significant Muc2 mRNA downregulation compared to the 3.75 mg/kg PE-MP-exposed group indicating a dose-related effect of PE-MP exposure. The outcomes of Alcian Blue staining, mucin area %, and goblet cells’ measurements were all analyzed and further illustrated that mucin secretion substantially declined following exposure to PE-MP. Consistent with these results, Jin et al. (2019) [9] and Chen et al. (2022) [22] also reported that polystyrene and polyvinyl chloride MP significantly diminished the mucus secretion, as well as Muc2’s mRNA levels. Conversely, Sun et al. (2021) [34] did not detect substantial changes in Muc2’s mRNA levels in mice colons after PE-MP exposure, and a trend of an increase was observed instead, although they reported a significant decrease in mucin production. They explained that this might be attributed to the upregulation feedback of reduced mucin.

In this study, melatonin administration significantly restored mucin depletion induced by PE-MP exposure, as demonstrated by the significant enhancement of goblet cells’ measurements and upregulation of Muc2 mRNA levels in the 3.75 mg/kg PE-MP + melatonin and 15 mg/kg PE-MP + melatonin groups compared to the 3.75 mg/kg PE-MP- and 15 mg/kg PE-MP-exposed groups, respectively. In accordance with our results, numerous studies have revealed that melatonin improves mucin production in the intestine. Kim et al. (2020) [29] demonstrated substantially elevated mRNA expression of Muc2 and induction of goblet cells after melatonin treatment. Lee et al. (2020) [38] also demonstrated that melatonin pretreatment onto goblet cells markedly enhanced mucin production and suggested that the molecular mechanism underlying this melatonin action is by acting on melatonin receptor two and inhibiting the Muc2 promoter hypermethylation in order to restore Muc2 production level in the intestinal epithelial cells. 

Maintaining intestinal homeostasis depends on intestinal epithelium’s structural integrity, maintained by junctional protein complexes (such as desmosomes, adherens junctions, and tight junctions) that seal adjacent epithelial cells and control intestinal permeability [39]. Tight junction function is crucial to intestinal health, since a compromised intestinal barrier may result in a variety of illnesses. Furthermore, this function is controlled by the tight junction proteins’ phosphorylation, distribution, and expression level [40].

Numerous animal investigations indicate that MP ingestion impairs critical intestinal functions, including gut barrier function, as well as gut microbiota modulation [6], although the primarily impaired barrier proteins remain undefined. Moreover, the MP that is usually investigated based on its in vivo impacts is polystyrene (PS) and polypropylene (PP), while PE, the most widely used type, is studied to a lesser extent. In this study, we examined the impact of PE-MP oral ingestion in albino rats on occludin, MLCK, claudin-1, and ZO-1 tight junction protein expressions. 

Our findings reveal that PE-MP exposure, in both the 3.75 and 15 mg/kg PE-MP-treated groups, significantly upregulated MLCK, downregulated occluding and ZO-1 mRNA, and decreased immunoreactivity of claudin-1. In contrast to the above results, Toto et al. (2022) [41] demonstrated substantially elevated mRNA levels of occludin and concentration of the ZO-1 protein in the duodenum of groups receiving PE-MP. Liang et al.’s (2021) [42] results were also different; they reported that PS50 and PS500 did not change tight junction protein mRNA expression in the jejunum, while when PS500, as well as PS50, were administrated as a mixture, most of the tight junction protein mRNA expression increased in the jejunum. The results of Qiao et al. (2021) [43] are, to some extent, in line with our results, as they revealed that nano PS-COOH moderately declined, whereas micro/nano PS-NH2 substantially diminished ZO-1 and claudin-1 expression. Conversely, micro PS-COOH and micro/nano PS exhibited a diminished effect on these protein expressions. Discrepancies in these results may be due to differences in the MP type, particle size, route, and duration of administration, which should be investigated in great detail. Studies that used PE-MP to determine the impact of this MP type on the tight junction protein are scarce; further studies using the same experimental conditions are needed in order to directly compare the results. 

Interestingly, melatonin treatment provided obvious intestinal barrier protection, as evidenced by its effect on mucin production and the intestinal epithelial tight junction proteins. Melatonin treatment significantly upregulated occludin and ZO-1 mRNA, claudin-1 protein expression, and downregulated MLCK mRNA in this study, which aligns with Lin et al. (2020) [44]. They demonstrated substantially elevated claudin-1, ZO-1, and occludin protein expression with melatonin treatment. 

The molecular mechanism underlying tight junction protein regulation seems complex and unclear; however, a substantial body of data has emphasized the significance of cytokines regulating numerous tight junction proteins. For example, IL-6, TNF-α, IL-10, IL-1β, and IL-17 were implicated in intestinal inflammation and regulation of tight junction proteins function [45]. 

We evaluated TNF-α and IL-1β cytokine levels to determine whether PE-MP exposure triggers inflammation and whether they are impacted in tight junction proteins regulation. 

Although both TNF-α and IL-1β are proinflammatory cytokines, PE-MP exerted a different effect on them. The results of this study reveal that the low dose (3.75 mg/kg) PE-MP did not induce significant changes in TNF-α compared to the controls. TNF-α is an essential mediator of inflammation in the gut, and its level is markedly elevated in patients with inflammatory conditions of the gut [46]. In addition, it has been shown to impair TJ expression or localization and, subsequently, induces barrier dysfunction [47]. Several previous studies did not reveal significant differences in the TNF-α level after exposure to various types of MP [48,49,50]. On the contrary, others reported an increase in TNF-α levels [51,52]. No in vivo studies reported significant decreases in the TNF-α value after MP exposure. However, the high dose (15 mg/kg) PE-MP administration in this study significantly decreased the TNF-α level compared to other groups. Notably, in Han et al.’s (2020) study, TNF-α released from peripheral blood mononuclear cells (PBMCs) declined as polyvinyl chloride (PVC) and acrylonitrile butadiene styrene (ABS) MP concentrations increased [50]. Further studies demonstrating the effect of various concentrations of MP on TNF-α release are needed. Gautam et al. (2022) also investigated the effects of PE-MPs on different human cell lines and reported that 5 µm PE-MPs reduced the level of TNFα production in THP-1 and U937 cell lines, while large-sized PE-MPs reduced the level of TNFα in U937 and upregulated it slightly in THP-1. They concluded that the immunomodulation caused by MPs could depend upon the nature of MPs or cells [53].

Our previous study demonstrated that MP exposure is linked to DNA hypermethylation and that the degree of hypermethylation increased with higher dose of exposure [54]. Further research is needed to investigate whether the TNF-alpha promoter is among the hypermethylated locations; this could explain the decreased TNF level observed with the high dose of PE-MP administration in this study. Furthermore, in this study, melatonin treatment with the high dose (15 mg/kg) PE-MP exposure restored TNF-α to levels close to the controls. Several studies have demonstrated the role of melatonin in modulating DNA methylation. It has been reported that melatonin significantly decreases the methylation level (5-mC) via downregulating the expression of DNA methyltransferases (DNMTs) [55], which further supports the assumption that MP-induced DNA hypermethylation could impact on decreasing TNF levels.

Although proinflammatory cytokines were frequently lowered by melatonin in many models of severe inflammation, contradictory responses were observed in some other circumstances where melatonin proved protective while elevating TNFα [33,56]. 

This study revealed a substantial elevation in IL-1β in the 3.75 and 15 mg/kg PE-MP-treated groups compared to the control. This finding further supports what several studies previously reported, demonstrating increased IL-1β expression after exposure to different types of MP [57,58,59]. The anti-inflammatory role of melatonin revealed in numerous studies [28,60,61,62] was evident in the 3.75 mg/kg PE-MP + melatonin and 15 mg/kg PE-MP + melatonin groups, as melatonin administration in these groups significantly decreased the IL-1β level.

Chemokines and cytokines are essential players in intestinal epithelial barrier integrity. Cytokines may enhance or diminish the permeability of epithelial tight junctions by altering the expression or distribution of their protein components. Cytokines may also stimulate myosin light-chain phosphorylation, causing tight junctions to contract and open [45]. Recent research has demonstrated that the IL-1β-induced increase in intestinal epithelial tight junction permeability in mouse small intestine, as well as Caco-2 intestinal epithelial monolayers, was mediated by the elevated MLCK gene activity and protein expression, along with elevated MLCK enzymatic activity. IL-1β-induced elevation in intestinal tight junction permeability is reliant on post-transcriptional degradation of occludin mRNA through an increase in miRNAs that bind to occludin mRNA 3’UTR, in addition to targeting the activation of MLCK gene activation [63,64,65]. Moreover, it has been shown that IL-1β disrupts the barrier function of Simian virus 40-immortalized human corneal epithelial (HCE) cells through ZO-1 and occludin redistribution from the borders of adjacent HCE cells in a manner reliant on the signaling pathways of NF-κB [66]. In addition, Maria-Ferreira et al. (2018) illustrated that IL-1β led to diminished claudin-1 expression [67].

Taken together, our results thus suggest that the increased IL-1β level observed with PE-MP exposure in this study may be impacted by the disruption of the barrier function through the upregulated MLCK mRNA, downregulated occludin and ZO-1 mRNA, and decreased claudin-1 expression. Moreover, TNF-α and IL-1β levels demonstrated in this study further suggested that epithelial barrier dysfunction is mediated mainly by increased IL-1β levels, as the TNF-α level did not change with a low dose of PE-MP and even significantly decreased with a high dose of PE-MP.

In this study, the significantly increased cleaved caspase-3-positive cells in the PE-MP-exposed groups further confirmed the increased apoptosis signs observed by EM examination. Several previous studies reported MP-induced apoptosis in various models [68,69]. Evidence has revealed that IL-1β is crucial for the process of apoptosis [70]. The increased IL-1β level observed with PE-MP exposure in this study suggests that the increased apoptosis observed may be IL-1β induced. Interestingly, melatonin treatment significantly decreased apoptotic cells and cleaved caspase-3 expression. Several previous studies reported the antiapoptotic effect of melatonin, which further support these results [71,72]. 

PE is one of the most prevalent and frequently utilized plastic types worldwide, and PE-MP rank as the most significant proportion of MP in the environment [34]. However, studies on PE-MP toxicological impact on the function of intestinal barriers are limited. Sun et al. (2021) [34] and Toto et al. (2022) [41] both investigated the effects of oral PE-MP on the intestinal barrier. However, PE exposure in both studies differed from that in the current study. Sun et al. (2021) [34] used MP with a size of 1–10 µm applied by oral gavage at a dose of 0.2 µg/g/day, while Toto et al. (2022) [41] used MP of 15–48 µm size in feed at a dose of 25 mg/day through 25 g feed. This is in contrast to our study, as we used MP with a size of 4–6 µm applied by oral gavage at a dose of 3.75 mg/kg/day (low-dose group) or 15 mg/kg/day ((high-dose group). Although Sun et al.’s (2021) [34] exposure design was relatively similar to ours, regarding the particle size and route of administration, the dose they used was much lower than ours, which may explain the absence of significant intestinal histological damage in their study. 

To conclude, the present study demonstrated that orally ingested PE-MP under the chosen experimental conditions provoked damaging effects on the intestinal mucus barrier, epithelial cells, and tight junction proteins primarily through decreased mucin secretion; downregulation of Muc2, occludin, and ZO-1 mRNA; and decreased claudin-1 protein expression. Higher concentration induced higher intestinal barrier disruption. Melatonin treatment provided obvious protection and significantly reduced PE-MP-induced intestinal barrier dysfunction. The impacts of MP ingestion on the intestinal barrier integrity are controversially discussed because of differences among the studies in several factors, such as the MP type, size of the particles, route, and duration administration. More research is required in order to address the existing knowledge gap on the detrimental effects of MP on intestinal barrier function.

## 4. Materials and Methods

### 4.1. Chemicals

PE-MP (MPP-635XF; Micro Powders Inc., Tarrytown, NY, USA) were used with mean particle sizes ranging from 4.0 to 6.0 µm. Characterization of the PE-MP particles was performed using scanning electron microscopy and documented in our previously published research [54]. Melatonin was purchased from Sigma-Aldrich (St. Louis, MI, USA). Chloroform (ADWIC, Abu-Zabaal, Qalyubia, Egypt), isopropanol (Middle East Chemicals MEC, Cairo, Egypt), 70% ethanol (the international company for medical industries, Giza, Egypt), and RNase-free Water (Vivantis technologies, Shah Alam, Malaysia) were used for RNA extraction. 

### 4.2. Animals

Forty-nine adult male albino rats (aged 8 weeks and weighing 150–180 g) were obtained from the Faculty of Science’s Animal House, Benha University, Egypt. Rats were categorized into seven groups of 7, and each group of rats was housed in separate cages, kept at 25 °C with a relative humidity of (45 ± 5%), and 12/12 h light and dark cycles. Additionally, all rats were provided with free access to water ad libitum, as well as food. Prior to the trial, all rats were acclimated to the housing environment of the animal facility for one week. The research adhered to the guidelines for the care and use of laboratory animals [73], as well as approved by the Faculty of Science’s Research Ethics Committee, Benha University, Egypt (approval no. ZD/FSc/BU-IACUC/2022-18b).

### 4.3. Study Design

All treatments were given by oral gavages for 5 weeks, and the animals were divided into seven groups of seven as follows:

Group I (control group; n = 14): Animals were equally subdivided into 2 groups (7 rats each):

Subgroup IA: This group received oral saline solution (0.9% NaCl) + ethanol (1%) once daily;

Subgroup IB: This group received 1 mL of oral corn oil once daily.

Group II (melatonin group; n = 7): This group was treated orally with immediately prepared melatonin (5 mg/kg/day). Melatonin was dissolved in absolute ethanol before being diluted with saline to achieve a final alcohol content of 1% ethanol. Following preparation, the bottles were protected from light utilizing aluminum foil and given to the animals [28,74]. 

Group III (low dose of PE-MP group; n = 7): This group received 3.75 mg of PE-MP/kg/day dissolved in 1 mL of corn oil [35].

Group IV (high dose of PE-MP group; n = 7): This group received 15 mg of PE-MP/kg/day dissolved in 1 mL of corn oil [35].

Group V (low dose of PE-MP + melatonin group; n = 7): This group received 3.75 mg of PE-MP/kg/day concurrently with melatonin (5 mg/kg/day). 

Group VI; (high dose of PE-MP+ melatonin group; n = 7): This group received 15 mg of PE-MP/kg/day concurrently with melatonin (5 mg/kg/day). 

At the end of the experiment, all rats were fasted for 8 h and euthanized via decapitation following inhalation anesthesia with isoflurane (El Amriya for pharmaceutical industries, Al Amyria, Alexandria). The intestinal tissue samples were collected on ice, and intestinal segments from the jejunum were cut.

### 4.4. Microplastics Quantification

PE-MP quantification in the jejunal tissue samples was conducted as per Hamed et al. (2019) [75]. In brief, the digestion of jejunal tissue samples (≈0.3 g) was conducted in hydrogen peroxide (10 mL) (30%, *v*:*v*) at 70 °C for 2 h. The resultant solution (100 µL) was subsequently examined and counted utilizing the light microscope.

### 4.5. Biochemical Analysis

Jejunal tissues samples were rinsed in ice-cold saline before being homogenized with phosphate buffer (pH 6–7) utilizing a Mixer Mill MM400 (Retsch, Germany). Centrifugation of the tissue homogenates was performed for 15 min at 10,000× *g*, 4 °C. Afterward, the supernatant was utilized to quantitively detect the following proinflammatory cytokines per the manufacturer: interleukin-1β (IL-1β) utilizing the rat IL-1β with the Quantikine ELISA Kit (Catalog #RLB00; R&D Systems, Inc., Minneapolis, MN, USA) and tumor necrosis factor (TNF-α) utilizing the rat TNF-alpha with the Quantikine ELISA Kit (Catalog #RTA00; R&D Systems, Inc., USA).

### 4.6. Quantitative Real-Time Polymerase Chain Reaction (qPCR) Analysis for mRNA Gene Expression of MUC2, Occludin, ZO-1, and MLCK

Samples of jejunal tissues utilized for PCR and RNA extraction were preserved in RNA later solution (RNA stabilizing reagent) (Qiagen Inc., Valencia, CA, USA) at 10 µL per 1 mg of tissue and subsequently kept at −80 °C until analysis.

#### 4.6.1. Total RNA Extraction and Reverse Transcription

Total RNA extraction from the jejunal tissue samples was performed using GENEzol™ Reagent (Catalog No: GZR100; Geneaid, New Taipei City, Taiwan) Plus chloroform, isopropanol, 70% ethanol, RNase-free Water, and microcentrifuge tubes (1.5 mL; RNase-free) based on the manufacturer’s instructions. RNA purity and concentration were evaluated by the measurement the absorbance at 280 nm and 260 nm utilizing a NanoDrop One spectrophotometer (Thermo Fisher Scientific, Waltham, MA, USA). The A260/A280 ratio of pure RNA ranges from 1.8 to 2.1 [76]. The RNA’s reverse transcription (RT) into complementary DNA (cDNA) was conducted in a Veriti™ Thermal Cycler (Applied Biosystems, Foster City, CA, USA) utilizing the TOPscript™ RT DryMIX (dT18/dN6) Kit (catalog no: RT220; enzynomics, Daejeon, Republic of Korea). Additionally, 5 μL RNA template and 20 μL nuclease-free water were added to each RT tube supplied. The temperature was fixed to 42 °C for 1 h, followed by 10 min of RTase inactivation at 85 °C.

#### 4.6.2. Quantitative Real-Time PCR

Gene expression’s relative quantitation was conducted utilizing Hera Sybr Green qPCR kit (Willowfort, UK). Singleplex reactions were performed; each mix contained 4 μL cDNA, 1 μL reverse primer (RP), 1 μL forward primer (FP), and 10 μL Hera Sybr master mix (2×), as well as up to 20 μL nuclease-free water. StepOne Real-Time Cycler (Applied Biosystem, Singapore) was utilized to run amplification. A 95 °C initial holding stage for 10 min was conducted prior to cycling for 40 cycles (15 s for denaturation at 95 °C and then annealing at 50 °C and extension for 1 min at 60 °C). In each run, a melting curve analysis was performed to confirm the assay specificity. The primer sequences are displayed in Table 1. After adjusting for B-actin expression, each sample’s mRNA expression was determined. The relative expression was computed utilizing the 2^−∆∆CT^ method [77]. The findings are expressed as the n-fold difference relative to controls.

### 4.7. Histological Study

Small jejunal parts approximately 1 cm long were fixed in a 10% neutral buffered formalin solution (10% NBF) for 24 h. Tissues were then processed for hematoxylin and eosin (H&E) stain. Moreover, other sections were stained with Alcian blue stain to study the goblet cells and the mucous layer according to Khedr et al. (2022) [78]. All these steps were conducted according to standard practice [79].

### 4.8. Immunohistochemical Study

Immunohistochemical staining was performed according to the standard method [80]. De-paraffinized retrieved jejunum tissue sections (5–7 μm) were treated for 20 min with 0.3% H2O2 before incubation with anti-claudin-1 (1:100—Thermofisher—37–4900) and anti-caspase-3 (active/pro) (1:200-Clone 31A1067, Catalog #MC0123, Medaysis, Livermore, CA, USA) overnight at 4 °C. Sections were then rinsed with PBS and treated with a secondary antibody HRP Envision kit (DAKO) for 20 min. Afterward, washing and incubation with diaminobenzidine (DAB) was conducted for 30 min at ambient temperature. Following the IHC reaction, dehydration of the slides in ethanol (70%, 96%, absolute), as well as xylene, was performed and subsequently closed with Dako Mounting Medium (Agilent, Santa Clara, CA, USA). The slides were washed in PBS before counterstaining with hematoxylin and then dehydrated, cleared in xylene, and covered for microscopic analysis.

### 4.9. Transmission Electron Microscopic Examination

The jejunal samples were divided into small pieces (1 mm^3^) for TEM examination before being fixed in buffered glutaraldehyde (2.5%) in phosphate buffer solution (0.1 M; pH 7.4) for 2 h at 4 °C. The samples were processed for ultrathin (70 nm) and semi-thin (0.5 um) sections in accordance with Ayub et al. (2017) [81]. At the electron microscope unit, the Regional Center for Mycology and Biotechnology (RCMB) at the Faculty of Science, Al-Azhar University (Cairo, Egypt), the examination and photographing of grids were conducted utilizing a transmission electron microscope (JEOL. JEM1010, Tokyo, Japan) at 70 kV. 

### 4.10. Imaging

Six nonoverlapping fields were randomly selected and scanned from each sample to measure mucin area and goblet cell number and diameter. The true positive reaction of the mucin was selected in each field/tissue sample. Then, the diameter of goblet cells was measured (minimum 30 goblet cells/field/tissue sample). Moreover, The area % of immunohistochemical mean claudin-1 and caspase-3 expression levels in the jejunum was also determined by randomly selecting and scanning six nonoverlapping fields. One standard module for assessing IHC expressions is the relative area % of positive reactions computed from the entire field area. All light microscopic examinations and data were collected utilizing an automated grade standard unit Leica Application module preprogrammed for histological analysis that was connected to a Full HD microscopic imaging system (Leica Microsystems GmbH, Wetzlar, Germany) to analyze the total sample section computed from the entire field area. All light microscopic examinations and data were collected utilizing an automated grade standard unit Leica Application module preprogrammed for histological analysis that was connected to a full HD microscopic imaging system (Leica Microsystems GmbH, Wetzlar, Germany) to analyze the total sample section.

### 4.11. Statistical Analysis

The 9th version of GraphPad Prism software (USA) was utilized for statistical analysis. The results are expressed as the mean ± standard deviation (SD). Normality was verified utilizing the Kolmogorov–Smirnov test. Moreover, comparisons among groups were analyzed utilizing one-way variance analysis (ANOVA) followed by Tukey’s multiple comparison tests. The statistical significance was determined at a *p*-value < 0.05. 

## Figures and Tables

**Table 1:** Primer sequences utilized for RT-PCR analysis.

Gene Name	Primer Sequences (5’→3’) (F: Forward; R: Reverse)	Accession Number
Occludin	F: ccttttgagagtccacct R: gtcttccgggtaaaaaga	AB016425.1
MLCK	F: gcacagaaatgggcaaac R: gcttcacaggtgtacttg	NM_001105874.2
ZO-1	F: tctgatcattccacacag R: tccactgctttgggtgta	NM_001106266
MUC2	F: acctggggtgacttccact R: atcaggacggactctatg	U68172.1
B-actin	F: ctacctcatgaagatcctcacc R: agttgaaggtagtttcgtggat	NM_007393.5

## Data Availability

All relevant raw data will be freely available by the authors.
